# Inhaled corticosteroids and the risk of type 2 diabetes among Swedish COPD patients

**DOI:** 10.1038/s41533-020-00207-7

**Published:** 2020-10-20

**Authors:** Björn Ställberg, Christer Janson, Karin Lisspers, Gunnar Johansson, Florian S. Gutzwiller, Karen Mezzi, Bine Kjoeller Bjerregaard, Anne Mette Tranberg Kejs, Leif Jorgensen, Kjell Larsson

**Affiliations:** 1 Department of Public Health and Caring Sciences, Family Medicine and Preventive Medicine, Uppsala University, Uppsala, Sweden; 2 Department of Medical Sciences: Respiratory, Allergy and Sleep Research, Uppsala University, Uppsala, Sweden; 3 Novartis Pharma AG, Basel, Switzerland; 4 IQVIA Solutions, Copenhagen, Denmark; 5 Integrative Toxicology, The National Institute of Environmental Medicine, Karolinska Institutet, Stockholm, Sweden

**Keywords:** Chronic obstructive pulmonary disease, Adverse effects

## Abstract

This study reports the association of ICS use and the risk of type 2 diabetes mellitus (T2DM) in Swedish patients with COPD using data from real-world, primary care settings. A total of 7078 patients with COPD were included in this analysis and the 5-year cumulative incidence rate per 100,000 person years was 1506.9. The yearly incidence rate per 100,000 person years ranged from 850 to 1919. Use of ICS especially at a high dose in patients with COPD was related to an increased risk of T2DM.

## Introduction

The effect of inhaled corticosteroids (ICSs) on the risk of diabetes in patients with chronic obstructive pulmonary disease (COPD) remains uncertain. Some studies suggest an increased risk of onset and progression of diabetes, specifically associated with high-dose ICSs^1–4^, while the other studies and reviews of controlled trials report no such association^5,6^. The Swedish national guidelines during the study period only recommended the use of ICS in combination with long-acting beta-2 agonists (LABA) for patients with COPD with a forced expiratory volume in 1 s <50% predicted and a history of exacerbations^7^. However, real-world studies indicate that ICSs/LABA are often used in Swedish patients in Global Initiative for Obstructive Lung Disease group A and B^8^ indicating that more patients could be exposed to the potential risks of ICS treatment than necessary. This study assessed the risk of type 2 diabetes mellitus (T2DM) associated with ICSs in Swedish patients with COPD.

## Results

### Study patients

A total of 7078 patients with COPD were included in this analysis and T2DM was reported in 418 (5.9%) patients. The 5-year cumulative incidence rate per 100,000 person years was 1506.9 and the yearly incidence rate per 100,000 person years ranged from 850 to 1919. The mean age of the total study population was 68.6 years and comprised of 55.7% females. Regarding the T2DM patients, the mean age was 67.4 years and 47.1% females. The follow-up time (in person-years) in the different ICS groups ranged from 365 to 11679 and from 11.5 to 464 in patients without T2DM and patients with T2DM, respectively. Among T2DM patients, the main ICS treatment groups were “no ICS stable dose” (*n* = 147), “low ICS stable dose” (*n* = 138), and “high ICS stable dose” (*n* = 35). Time to T2DM diagnosis was not statistically different across the groups.

### Risk of type 2 diabetes

For almost all groups, except the low-dose ICS groups with mixed ICS dosage and decrease or increase in the ICS dosage, a significant increased risk of T2DM was observed compared to the reference population. A dose-related association was observed as the risk of T2DM was 32 and 64% higher among patients using stable continuous low- and high-dose ICS over the whole study period, respectively, compared to the reference controls using no ICS. Furthermore, for the patients who were using high-dose ICS, the risk of T2DM was 100% (increased usage) and 96% (mixed usage) higher compared to the reference group (Table 1). Further, adjustment for hypertension (I10) and/or heart failure (I50), did not significantly affect the results (data not shown).

**Table 1:** Association between ICS therapy for COPD and risk of type 2 diabetes mellitus.

ICS and treatment pattern	Number of T2DM patients	Time (years) to T2DM		RR, adjusted for BMI, time since COPD, sex, and age	
		Median (range)	Mean (SD)	RR (95% CI)	*p* value
No ICS^a^, at any time point^b^	147	1.6 (0.2–8.2)	2.4 (1.9)	1 (Reference)	—
No ICS^a^, usage decrease^b^	24	1.7 (1.1–5.9)	2.4 (1.5)	1.61 (1.03–2.52)	0.0350
No ICS^a^, usage mix^b^	19	6.6 (2.7–9.8)	6.3 (1.9)	1.65 (1.00–2.71)	0.0493
Low ICS^a^, usage decrease^b^	3	1.6 (1.1–1.7)	1.5 (0.3)	0.52 (0.17–1.65)	0.2696
Low ICS^a^, usage increase^b^	5	1.7 (1.4–4.6)	2.3 (1.3)	0.52 (0.21–1.28)	0.1556
Low ICS^a^, usage mix^b^	18	4.2 (2.9–8.5)	4.7 (1.6)	0.81 (0.49–1.35)	0.4239
Low ICS^a^, usage stable^b^	138	2.1 (0.3–8.2)	2.5 (1.8)	1.32 (1.04–1.67)	0.0201
High ICS^a^, usage increase^b^	16	2.1 (1.2–8.7)	2.9 (2.1)	2.00 (1.19–3.37)	0.0088
High ICS^a^, usage mix^b^	13	4.1 (2.6–6.5)	4.0 (1.1)	1.96 (1.10–3.51)	0.0234
High ICS^a^, usage stable^b^	35	1.3 (0.3–6.7)	2.0 (1.7)	1.64 (1.13–2.38)	0.0088

*BMI* body mass index, *CI* confidence interval, *COPD* chronic obstructive pulmonary disease, *ICS* inhaled corticosteroids, *RR* relative risk, *SD* standard deviation, *T2DM* type 2 diabetes mellitus.

^a^ICS dosage at the time of T2DM diagnosis, death, or end of follow-up (whichever comes first).

^b^Treatment pattern from index date to event (T2DM, death, end of follow-up).

These results confirm previous studies that exposure of ICS in COPD was associated with an increased risk of onset of diabetes^2,4,9–11^.

## Methods

### Study design and patients

We analysed data from ARCTIC, a real-world observational study of Swedish primary care patients with COPD^12–15^. Ethical approval for the study was obtained from the local Ethical Regional Board in Uppsala, Sweden on 11 December 2014 (number: 2014-397) for accessing the National Health Register and for recruiting primary care centres to the study. An amendment specifying additional analysis was approved by the Ethical Regional Board in Uppsala on 6 October 2017. Data from all records were de-identified, and therefore, patient consent was not required by the ethics committee.

Following ethical approval, electronic medical record (EMR) data linked to National Health Registries were collected from COPD patients in 52 Swedish primary care centres (2000–2014). The study population consisted of patients aged ≥40 years who had received a physician’s diagnosis of COPD (International Classification of Diseases, Tenth Edition (ICD-10) code: J44), with or without asthma (ICD-10 code: J45/J46) in a primary care setting (EMR database) or in a hospital setting (according to the National Patient Register). The information on ICS usage might be incomplete in the case notes from the general practitioners. Hence, for this analysis, the study population was limited to the patients diagnosed with COPD since 01 August 2005 as the national drug prescription register with full details of all dispensed medications was operational from July 2005. In order to focus exclusively on the effects of ICS, patients with more than one dispensation of oral corticosteroids (OCSs) or patients who received more than a normal pack, i.e. 25 tablets with 5 mg prednisolone or equivalent per tablet at any time point from 1 year prior to index and during the whole study period were excluded. We excluded 1490 patients due to OCSs, ending up with 7078 patients in the study population.

### Statistical analyses

The study index date constituted the time of the first recorded physician’s diagnosis of COPD during the enrolment timeframe. In the Poisson regression model, the follow-up time from COPD index to event, i.e. either incidence of T2DM (ICD code E11 or E14, in EMR or inpatient/outpatient register, or if the patient had filled a prescription of TD2M drug, i.e. ATC code A10B) or end of study, whichever comes first was split into equally sized intervals of 365 days (the last time interval will be <365 days). Body mass index (BMI) was assessed within each of these time intervals plus allowing for 6 months of the next time interval (e.g., for the first year of follow-up, BMI was assessed from COPD diagnosis and going 18 months forward). The treatment pattern of ICS dosage over time varied among COPD patients, hence we have categorised the ICS treatment pattern during the study period into (i) stable continuous dosage during the whole study period, i.e. no ICS, low-dose ICS, and high-dose ICS; (ii) mixed ICS dosage; (iii) decrease in the ICS dosage, and (iv) increase in the ICS dosage. A patient is assigned mixed ICS dosage if the previous levels of ICS have been varying between the three main ICS categories (no ICS, low-dose ICS, and high-dose ICS). The decreased/increased ICS dosage is assigned if the patient is switching from high dose to low/no ICS dose or vice versa and maintaining the same direction of treatment pattern, i.e. decreasing or increasing. The reference population considered in the analysis are “no ICS stable dose at any time point” COPD patients. The dispensed amounts of different types of ICS (budesonide, fluticasone propionate, beclomethasone, rest of the R03BA group, and all steroid combinations in the R03AK group) were converted to budesonide equivalents. The average daily dosage of ICS was calculated based on the filled prescriptions in the given time interval and the patients were stratified by the level of ICS exposure after the index date until an event (high dose: ≥640 μg/day budesonide or equivalent; low dose: <640 μg/day budesonide or equivalent; and no ICS).

The last time interval of follow-up might vary in duration and a shorter follow-up period (<180 days) can result in misleading information on ICS dosage. To avoid this, the dosage for the last time interval was calculated based on the prescriptions of ICS received 6 months before the last follow-up interval. Patients without any follow-up or patients who have filled the first prescription within 3 months prior to the event were excluded from the analysis. Poisson regression was used to model the impact of ICS on the risk of T2DM among COPD patients (time to event). As the exposure time was split into equally sized intervals, the constant rates were assumed within the time interval. The analysis was presented as relative risk with 95% confidence interval adjusted for BMI, time since COPD, sex, and age. The statistical analysis software used was “PC SAS for Windows” Version 9.4 (SAS Institute Inc., Cary, NC).

### Strengths and limitations

The present study has several important strengths. The real-world study design and the large sample size of high-quality national registry data from primary care settings adequately reflect the general population and clinical practice in Sweden. Nevertheless, this study also has certain limitations. The retrospective study design introduces the potential for bias and confounding due to variables that may not have been accounted for in our analysis. Although all patients had physician-diagnosed COPD, the accuracy of COPD diagnoses and the severity of disease could not be verified, as spirometry data were not available in many patients. Due to lack of spirometry data, it was not possible to assess the impact of disease severity on the incidence of T2DM. This study was conducted only in Swedish patients; it is therefore uncertain whether these findings can be extrapolated to a more diverse group of patients and to other healthcare systems.

Hence, we conclude that patients with COPD who initiate treatment with ICS, especially with high dose, may have an elevated risk of developing T2DM.

### Reporting summary

Further information on research design is available in the Nature Research Reporting Summary linked to this article.

## Supplementary information

Reporting Summary

## Data Availability

The data for this study were obtained from EMRs in primary care and the Swedish National Health Register. Restrictions apply to the availability of these data, which were used under license for the current study, and so are not publicly available. Data are, however, available from IQVIA Solutions, Copenhagen, Denmark upon reasonable request and with permission of the Swedish National Health Register.
