# Supplementary material for: Inhaled corticosteroids and the risk of type 2 diabetes among Swedish COPD patients
Source: NPJ Prim Care Respir Med. 2020 Oct 20;30:47. doi: 10.1038/s41533-020-00207-7 (PMC7576803; doi:10.1038/s41533-020-00207-7)
Supplement: Supplementary file 1 — Reporting Summary [file 41533_2020_207_MOESM1_ESM.pdf]

nature research

Corresponding author(s): Björn Ställberg

Last updated by author(s): Sep 23, 2020

Reporting Summary

Nature Research wishes to improve the reproducibility of the work that we publish. This form provides structure for consistency and transparency in reporting. For further information on Nature Research policies, see our Editorial Policies and the Editorial Policy Checklist.

Statistics

For all statistical analyses, confirm that the following items are present in the figure legend, table legend, main text, or Methods section.

n/a	Confirmed	
☐	☒	The exact sample size (n) for each experimental group/condition, given as a discrete number and unit of measurement
☐	☒	A statement on whether measurements were taken from distinct samples or whether the same sample was measured repeatedly
☐	☒	The statistical test(s) used AND whether they are one- or two-sided<br>*Only common tests should be described solely by name; describe more complex techniques in the Methods section.*
☐	☒	A description of all covariates tested
☐	☒	A description of any assumptions or corrections, such as tests of normality and adjustment for multiple comparisons
☐	☒	A full description of the statistical parameters including central tendency (e.g. means) or other basic estimates (e.g. regression coefficient) AND variation (e.g. standard deviation) or associated estimates of uncertainty (e.g. confidence intervals)
☒	☐	For null hypothesis testing, the test statistic (e.g. F, t, r) with confidence intervals, effect sizes, degrees of freedom and P value noted<br>*Give P values as exact values whenever suitable.*
☒	☐	For Bayesian analysis, information on the choice of priors and Markov chain Monte Carlo settings
☒	☐	For hierarchical and complex designs, identification of the appropriate level for tests and full reporting of outcomes
☒	☐	Estimates of effect sizes (e.g. Cohen's d, Pearson's r), indicating how they were calculated

Our web collection on statistics for biologists contains articles on many of the points above.

Software and code

Policy information about availability of computer code

Data collection	Electronic medical record (EMR) data were collected using an established software system (Pygargus Customized eXtraction Program [CXP])
Data analysis	The statistical analysis software used was ‘PC SAS for Windows’ Version 9.4.

For manuscripts utilizing custom algorithms or software that are central to the research but not yet described in published literature, software must be made available to editors and reviewers. We strongly encourage code deposition in a community repository (e.g. GitHub). See the Nature Research guidelines for submitting code & software for further information.

Data

Policy information about availability of data

All manuscripts must include a data availability statement. This statement should provide the following information, where applicable:

- Accession codes, unique identifiers, or web links for publicly available datasets
- A list of figures that have associated raw data
- A description of any restrictions on data availability

The data for this study were obtained from EMRs in primary care and the Swedish National Health Register. Restrictions apply to the availability of these data, which were used under license for the current study, and so are not publicly available. Data are however available from IQVIA Solutions, Copenhagen, Denmark, upon reasonable request and with permission of the Swedish National Health Register.

Field-specific reporting

Please select the one below that is the best fit for your research. If you are not sure, read the appropriate sections before making your selection.

☒ Life sciences ☐ Behavioural & social sciences ☐ Ecological, evolutionary & environmental sciences

For a reference copy of the document with all sections, see nature.com/documents/nr-reporting-summary-flat.pdf

Life sciences study design

All studies must disclose on these points even when the disclosure is negative.

Sample size	ARCTIC is a large, real-world, retrospective cohort study conducted in 18,586 Swedish COPD patients and a matched reference population from 52 primary care centers in 2000–2014.
Data exclusions	Not applicable
Replication	This was a retrospective analysis and no experimental methods were setup. Data collection and analysis were the main aspects, which were repeated for all included patients from 2000–2014
Randomization	Not applicable
Blinding	Not applicable

Reporting for specific materials, systems and methods

We require information from authors about some types of materials, experimental systems and methods used in many studies. Here, indicate whether each material, system or method listed is relevant to your study. If you are not sure if a list item applies to your research, read the appropriate section before selecting a response.

Materials & experimental systems

n/a	Involved in the study	
☒	☐	Antibodies
☒	☐	Eukaryotic cell lines
☒	☐	Palaeontology and archaeology
☒	☐	Animals and other organisms
☐	☒	Human research participants
☒	☐	Clinical data
☒	☐	Dual use research of concern

Methods

n/a	Involved in the study	
☒	☐	ChIP-seq
☒	☐	Flow cytometry
☒	☐	MRI-based neuroimaging

Human research participants

Policy information about studies involving human research participants

Population characteristics	The study population consisted of patients aged ≥ 40 years who had received either a physician's diagnosis of COPD (ICD-10 code: J44) in primary care (EMR database) or a physician's diagnosis of asthma (ICD-10 code: J45/J46) in primary care that was later verified as COPD, or COPD was added to the asthma diagnosis in the hospital setting according to the National Patient Register.
Recruitment	EMR data for COPD patients between 2000 and 2014 from 52 primary care centres across Sweden were collected. EMR data were linked by the Swedish National Board of Health and Welfare using individual patient identification (ID) numbers to National Registry data sources (patient IDs were pseudonymized). These data sources included: (i) the Longitudinal Integration Database for Health Insurance and Labour Market Studies (LISA), which contains socio-demographic data, including educational level, marital status and family situation, occupational status, retirement and economic compensation and social benefits; (ii) the National Patient Register, which contains data related to diagnosis from secondary care (ICD-10 code and associated position), including surgery, gender, age, region, hospital visits, specialty visits, hospital admissions, discharges, medical procedures and surgeries performed in inpatient and outpatient specialist settings; (iii) the National Prescription Register (from 2005), which contains the full details of all dispensed medications (ATC codes) from both primary and secondary care, including brand name, prescription date, dose, strength, pack size, specialty of the prescriber and costs associated with the drug prescription; and (iv) the Cause of Death Register, which holds information related to sex, date of death and the underlying cause of death.
Ethics oversight	Ethical approval for the study was obtained from the local Ethical Regional Board in Uppsala, Sweden, on 11 December 2014 (number: 2014-397), for accessing the National Health Register and for recruiting primary care centres to the study.

Note that full information on the approval of the study protocol must also be provided in the manuscript.
